# The Role of Dorsal Raphe Serotonin Neurons in the Balance between Reward and Aversion

**DOI:** 10.3390/ijms21062160

**Published:** 2020-03-21

**Authors:** Yuma Nagai, Kaito Takayama, Naoya Nishitani, Chihiro Andoh, Masashi Koda, Hisashi Shirakawa, Takayuki Nakagawa, Kazuki Nagayasu, Akihiro Yamanaka, Shuji Kaneko

**Affiliations:** 1 Department of Molecular Pharmacology, Graduate School of Pharmaceutical Sciences, Kyoto University, Kyoto 606-8501, Japan; nagai@mol.pharm.kyoto-u.ac.jp (Y.N.); takayama@mol.pharm.kyoto-u.ac.jp (K.T.); chihiroa@mol.pharm.kyoto-u.ac.jp (C.A.); koda@mol.pharm.kyoto-u.ac.jp (M.K.); shirakaw@pharm.kyoto-u.ac.jp (H.S.); 2 Department of Neuropharmacology, Faculty of Medicine and Graduate School of Medicine, Hokkaido University, N15 W7 Kita-ku, Sapporo 060-8638, Japan; nishitani@med.hokudai.ac.jp; 3 Department of Clinical Pharmacology and Therapeutics, Kyoto University Hospital, 54 Shogoin Kawahara-cho, Sakyo-ku, Kyoto 606-8507, Japan; tknakaga@kuhp.kyoto-u.ac.jp; 4 Department of Neuroscience II, Research Institute of Environmental Medicine (RIEM), Nagoya University, Nagoya 464-8601, Japan; yamank@riem.nagoya-u.ac.jp

**Keywords:** serotonin, optogenetics, reward

## Abstract

Background: Reward processing is fundamental for animals to survive and reproduce. Many studies have shown the importance of dorsal raphe nucleus (DRN) serotonin (5-HT) neurons in this process, but the strongly correlative link between the activity of DRN 5-HT neurons and rewarding/aversive potency is under debate. Our primary objective was to reveal this link using two different strategies to transduce DRN 5-HT neurons. Methods: For transduction of 5-HT neurons in wildtype mice, adeno-associated virus (AAV) bearing the mouse tryptophan hydroxylase 2 (TPH2) gene promoter was used. For transduction in Tph2-tTA transgenic mice, AAVs bearing the tTA-dependent TetO enhancer were used. To manipulate the activity of 5-HT neurons, optogenetic actuators (CheRiff, eArchT) were expressed by AAVs. For measurement of rewarding/aversive potency, we performed a nose-poke self-stimulation test and conditioned place preference (CPP) test. Results: We found that stimulation of DRN 5-HT neurons and their projections to the ventral tegmental area (VTA) increased the number of nose-pokes in self-stimulation test and CPP scores in both targeting methods. Concomitantly, CPP scores were decreased by inhibition of DRN 5-HT neurons and their projections to VTA. Conclusion: Our findings indicate that the activity of DRN 5-HT neurons projecting to the VTA is a key modulator of balance between reward and aversion.

## 1. Introduction

Reward processing is fundamental for animals to survive and reproduce. Animal and human behaviors are reinforced by rewards; the disruption of this reward-related neuronal circuit is one of the causes of psychiatric disorders including major depression and schizophrenia in humans [1,2,3,4,5]. Studies have demonstrated that dopamine (DA) neurons in the midbrain ventral tegmental area (VTA) play a key role in reward processing and motivation [6,7]. In addition to DA neurons, many reports have suggested that serotonin (5-HT) neurons play a pivotal role in reward processing [8,9,10,11]. Intracranial self-stimulation of the dorsal raphe nucleus (DRN), the largest serotonergic nucleus, is sufficient for reinforcement learning [12,13,14,15]. Neuronal activity of a part of DRN neurons is positively or negatively correlated with reward size [16,17]. Pharmacological elevation of extracellular 5-HT levels by selective serotonin reuptake inhibitors (SSRIs) and 5-HT1A receptor antagonists attenuated the decrease of interest during withdrawal from nicotine or amphetamine [18]. The rewarding effect of cocaine was observed in DA transporter-knockout mice or dopamine-deficient mice, which was, at least partly, mediated by the 5-HT transporter [19,20]. Moreover, pharmacological analyses have revealed that the activity of mesencephalic DA neurons is modulated by 5-HT [21,22]. These reports strongly suggest that DRN 5-HT neurons play a critical role in reward processing.

The DRN contains a large number of 5-HT neurons that project to the forebrain and midbrain, including reward-related brain areas, such as the VTA, nucleus accumbens (NAc), and lateral hypothalamus (LH) [23,24,25]. Previous reports have indicated the importance of 5-HT neuronal signaling in the pathophysiology and therapeutics of major depressive disorders [26,27,28,29]. Indeed, previous reports have demonstrated that the activation of DRN 5-HT neurons induces an antidepressant-like effect in mice and rats [30,31]. Similar to an antidepressive effect, rewarding potency of DRN 5-HT neurons has been studied by using several lines of genetically-modified mice [9,10,11,32,33,34,35]. Liu et al. and Li et al. have shown that sucrose intake activates DRN 5-HT neurons and that the activation of DRN 5-HT neurons causes operant reinforcement using ePet1-Cre mice [9,10]. In contrast, McDevitt et al. reported that stimulation of DRN 5-HT neurons failed to reinforce nose-poke behaviors by using the same Cre-driver as Liu et al. and Li et al [32]. Moreover, Fonseca et al. have shown similar results using SERT-Cre mice [34]. However, Wang et al. have recently demonstrated that stimulation of 5-HT projection from the DRN to the VTA induces a rewarding effect via the activation of glutamate and 5-HT receptors by using SERT-Cre mice [11]. Collectively, the rewarding potency of the activation of DRN 5-HT neurons is under debate. Moreover, the consequence of inhibition of DRN 5-HT neurons on reward/aversive potency remains to be elucidated.

To address these issues, in the present study, we examined the link between the excitation of DRN 5-HT neurons and reward potency, and that between inhibition of DRN 5-HT neurons and aversive potency using two different methods selectively transducing 5-HT neurons. We examined the rewarding potency of optogenetic manipulation of DRN 5-HT neurons using an adeno-associated virus (AAV) expressing optogenetic actuators under the control of the mouse tryptophan hydroxylase 2 (TPH2) promoter [31]. We also employed the Tph2-tTA transgenic mouse line [36] in combination with tTA-dependent AAVs, to further investigate the reward/aversive potency of these neurons. We found that the optogenetic activation of DRN 5-HT neurons and 5-HT projections from the DRN to VTA strongly reinforced nose-poke self-stimulation behavior and induced conditioned place preference, using an AAV bearing the TPH2 promoter. Consistent with these results, the optogenetic activation and inhibition of DRN 5-HT neurons using the Tph2-tTA driver produced reward and aversive effects, respectively. These results strongly suggest that the activity of DRN 5-HT neurons plays a critical role in reward processing.

## 2. Results

### 2.1. Effect of Optogenetic Activation of DRN 5-HT Neurons Transduced by AAV Bearing Mouse TPH2 Promoter in Nose-Poke Self-Stimulation Test and Conditioned Place Preference (CPP) Test

**Figure 1:**
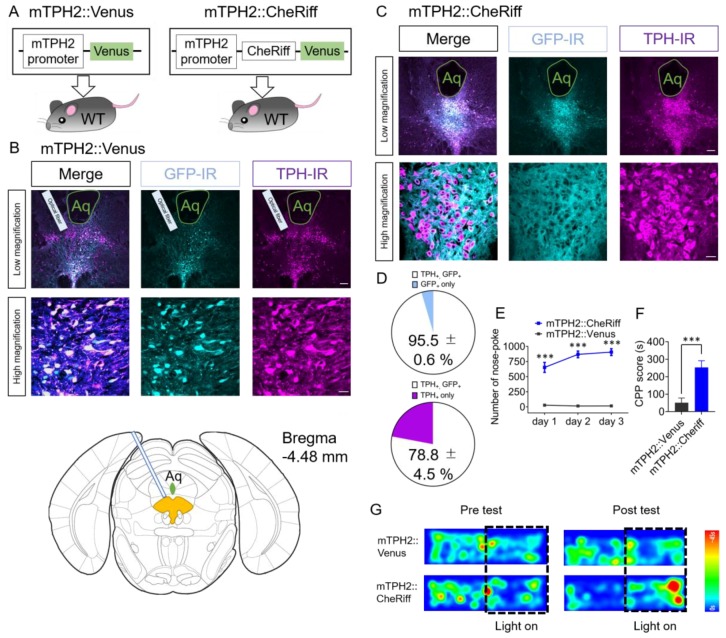
Stimulation of the dorsal raphe nucleus (DRN) serotonin (5-HT) neurons transduced by an adeno-associated virus (AAV) bearing the mouse tryptophan hydroxylase 2 (TPH2) promoter induces self-stimulation behavior and conditioned place preference. (**A**) Schematic of the experiment. (**B**,**C**) Four weeks after injection, coronal sections containing the dorsal raphe nucleus (DRN) were prepared and stained by anti-green fluorescent protein (GFP) and anti-tryptophan hydroxylase (TPH) antibodies. Stained sections were imaged using confocal microscopy. Scale bars = 100 μm (low magnification), 20 μm (high magnification). Aq, Aqueduct. (B, bottom panel) Drawings of the coronal section including injection site from the Atlas. DRN is colored in yellow. (**D**) The number of TPH- and GFP-immunoreactive cells in mTPH2::Venus injected animals were counted. (Top) Data represent mean ± SEM of the percentage of double-positive cells in GFP-positive cells. (Bottom) Data represent mean ± SEM of the percentage of double-positive cells in TPH-positive cells. *n* = 4 mice. (**E**) After injection of mTPH2::Venus or mTPH2::CheRiff, the number of nose-poke responses in the operant chamber was measured. Blue light (20 Hz, 10 ms duration, 20 pulses, 5 mW) was delivered when mice performed nose-poke responses. Data represent mean ± SEM. *n* = 7 (Venus), 15 (CheRiff) mice. *** *p* < 0.001 vs. Venus (Day 1; mTPH2::Venus, 28.9 ± 2.3, mTPH2::CheRiff, 652.7 ± 82.5, Day 2; mTPH2::Venus, 15.9 ± 4.8, mTPH2::CheRiff, 868.4 ± 55.1, Day 3; mTPH2::Venus, 17.1 ± 4.9, mTPH2::CheRiff, 906.5 ± 55.9; two-way repeated-measures ANOVA; Interaction, F(2, 40) = 6.59, *p* = 0.0034, Time, F(1.218, 24.35) = 5.37, *p* = 0.024, Opto, F(1, 20) = 83.84, *p* < 0.001, Subject, F(20, 40) = 7.08, *p* < 0.001; Bonferroni posttests; Days 1–3, *** *p* < 0.001). (**F**) In the conditioned place preference (CPP) test, spent time in the chamber associated with blue light illumination to the DRN was examined. Data represent mean ± SEM. *n* = 9 mice (Venus, CheRiff). *** *p* < 0.001 vs. Venus. (mTPH2::Venus, 51.39 ± 26.53 s, mTPH2::CheRiff, 253.2 ± 38.2 s; unpaired *t*-test; *p* = 0.0005). (**G**) Representative heat map of mouse movement during the pretest at Day 1 and posttest at Day 4. WT, wild type; IR, immunoreactivity.

We and others previously reported that lentiviral vectors bearing the mouse and rat TPH2 promoter can selectively transduce 5-HT neurons in mice and rats, respectively [31,37]. To manipulate 5-HT neuronal activity not only at the cell soma region but also at the axon terminal region, we made an adeno-associated virus (AAV) bearing CheRiff, an optogenetic actuator that shows good expression and membrane trafficking [38], under the control of the mouse TPH2 promoter. The specificity of the promoter was immunohistochemically examined in AAV-mTPH2-Venus-WPRE (mTPH2::Venus) injected mice. We found that 95.5% ± 0.6% of Venus immunoreactive cells were also immunoreactive for TPH2 in the DRN (*n* = 4 mice, Figure 1A–D). Moreover, 78.8% ± 4.5% of TPH2-immunoreactive cells were also immunoreactive for Venus (*n* = 4 mice, Figure 1D). To investigate the effect with the activation of DRN 5-HT neurons on operant and Pavlovian conditioning in these mice, we performed the nose-poke self-stimulation test and the conditioned place preference (CPP) test (Figure 1E–G). Four weeks after injection of AAV-mTPH2-Cheriff-EGFP-WPRE (mTPH2::CheRiff) or mTPH2::Venus, mice were placed in an operant chamber equipped with one active nose-poke port. When mice performed nose-poke responses into the active port, a set of light pulses (20 Hz, 10 ms duration, 20 pulses, 5 mW at the fiber tip) were delivered into the DRN through implanted fiber-optic. We found that mTPH2::CheRiff mice showed significantly more nose-poke responses than mTPH2::Venus control mice for three consecutive days (Figure 1E, Supplementary movie 1, 2). In the CPP test, mice were allowed to freely explore two conditioning chambers which had different colored walls and different textured floors at Day 1. At Days 2 and 3, mice were confined to one chamber that was initially preferred for 20 min without light illumination. After an interval of more than 4 h, mice were confined to another chamber (i.e., the chamber that was initially with unpreferred) for 20 min with light illumination. At Day 4, mice were again allowed to freely explore two conditioning chambers and the difference in spent time in the chamber associated with light illumination between Day 1 and Day 4 (Day 4–Day 1; CPP score) was measured. Different cohort of mice was injected with mTPH2::CheRiff and mTPH2::Venus. Mice injected with mTPH2::CheRiff showed significantly higher CPP score than mTPH2::Venus control (Figure 1F,G). Moreover, two-way repeated-measures ANOVA of spent time in the chamber associated with light stimulation in Days 1 and 4 revealed the significant interaction between time and AAVs (F(1, 16) = 18.85, *p* < 0.001, Supplementary Figure S1A). These results indicate that the activation of the DRN 5-HT neurons, transduced by an AAV bearing the mTPH2 promoter, was sufficient to elicit self-stimulation behavior and place preference.

### 2.2. Optogenetic Activation of 5-HT Neuron Terminals in the VTA is Responsible for Reinforcement of Nose-Poke Behavior but not in the LH, CeA, NAc, or Ventral Pallidum (VP)

**Figure 2:**
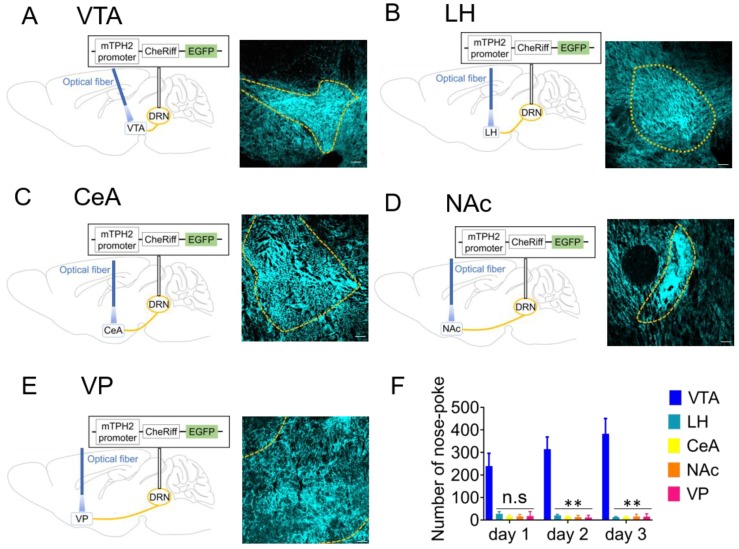
Optogenetic activation of 5-HT neuron terminals in ventral tegmental area (VTA) is responsible for inducing self-stimulation behavior. (**A**–**E**) (left) Schemas showing the sites of adeno-associated virus injection and fiber implantation. (right) Four weeks after mTPH2::CheRiff injection in the dorsal raphe nucleus (DRN), coronal sections containing the ventral tegmental area (VTA) (**A**), lateral hypothalamus (LH) (**B**), central amygdala (CeA) (**C**), nucleus accumbens (NAc) (**D**), and ventral pallidum (VP) (**E**) were prepared and stained by anti-green fluorescent protein (GFP) antibodies. Stained sections were imaged using confocal microscopy. Scale bars = 100 μm. (**F**) After injection of mTPH2::CheRiff, the number of nose-poke responses in the operant chamber was measured. Blue light was unilaterally delivered when mice performed nose-poke responses. Data represent mean + SEM of the number of nose-pokes during a 30 min session. VTA; *n* = 8 mice, LH; *n* = 7 mice, CeA; *n* = 8 mice, NAc; *n* = 8 mice, VP; *n* = 7 mice. ** *p* < 0.01 vs. VTA, *n*.s.: not significant (Day 1; VTA, 240.1 ± 56.1, LH, 28.3 ± 9.4, CeA, 17.0 ± 5.7, NAc, 17.4 ± 2.8, VP, 17.6 ± 7.6, Day 2; VTA, 314.6 ± 53.9, LH, 21.1 ± 5.0, CeA, 14.9 ± 3.3, NAc, 13.6 ± 2.8, VP, 13.6 ± 2.8, Day 3; VTA, 382.9 ± 67.7, LH,14.0 ± 2.2, CeA, 13.9 ± 3.6, NAc, 16.5 ± 3.4, VP 16.5 ± 3.4; two-way repeated-measures ANOVA; Interaction, F(8, 66) = 6.298, *p* < 0.001, Time, F(1.492, 49.23) = 4.118, *p* = 0.0326, Area, F(4, 33) = 26.40, *p* < 0.001; Bonferroni posttests; Day 1, LH; *p* = 0.0672, CeA; *p* = 0.0526, NAc; *p* = 0.0536, VP; *p* = 0.0526 vs. VTA, Day 2, LH; *p* = 0.0093, CeA; *p* = 0.0084, NAc; *p* = 0.0082, VP; *p* = 0.0079 vs. VTA, Day 3, LH; *p* = 0.0095, CeA; *p* = 0.0094, NAc; *p* = 0.0099, VP; *p* = 0.0096 vs. VTA). mTPH2, mouse tryptophan hydroxylase 2.

DRN 5-HT neurons project to a variety of brain areas including the VTA, LH, CeA, NAc, and ventral pallidum (VP) [23,39,40]. We, therefore, examined which projections from the DRN are responsible for reward processing. Four weeks after injection of mTPH2::CheRiff, we observed strong GFP expression in the VTA, LH, CeA, NAc, and VP, indicating that 5-HT terminals in these regions express sufficient amount of CheRiff (Figure 2A–E, Supplementary Figure S2). Fiber-optics were implanted in these regions to stimulate the axon terminals, and the effect of blue light application in the nose-poke test was investigated. Mice with implanted fiber into the VTA showed significantly more nose-poke responses than those into other brain regions (Figure 2F). Moreover, the number of nose-poke responses in mTPH2::Venus mice with implanted fiber into the VTA was far less than that in mTPH2::CheRiff mice (Supplementary Figure S3). These results suggest that among the diverse 5-HT projections from the DRN, VTA-projecting 5-HT neurons play a key role in inducing self-stimulation behavior.

### 2.3. Effect of Optogenetic Activation of DRN 5-HT Neurons Targeted by Tph2-tTA Transgenic Mice in Nose-Poke Self-Stimulation Test and CPP Test

**Figure 3:**
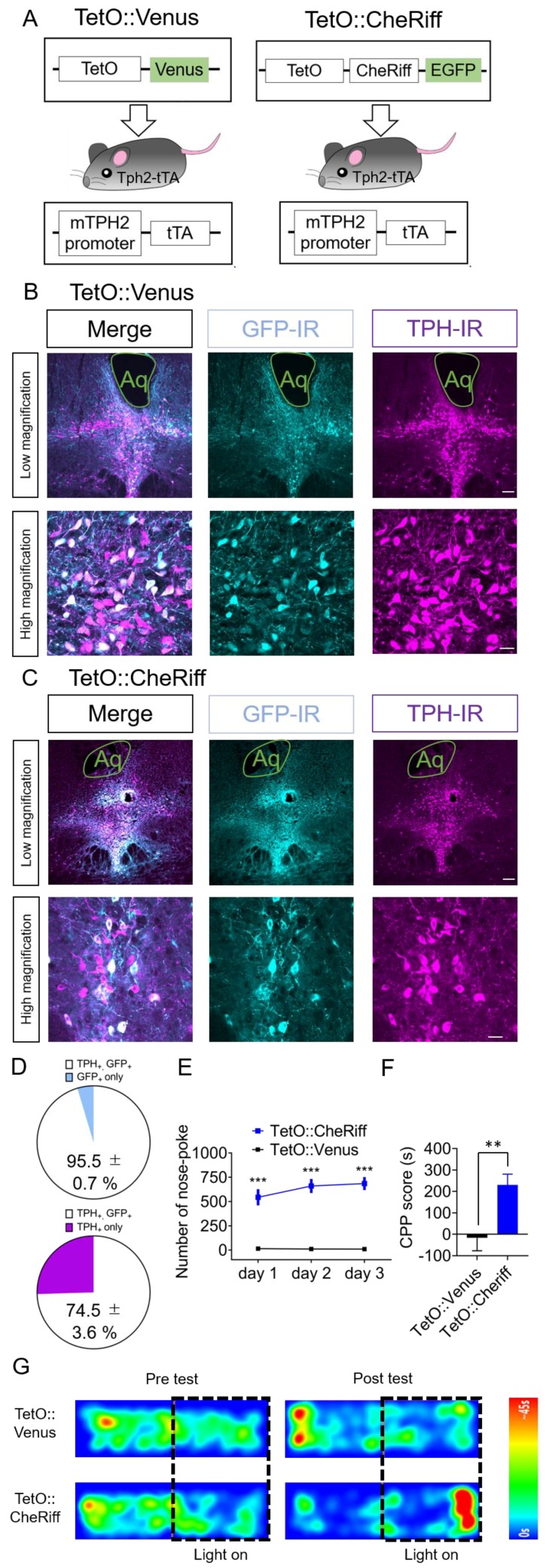
Effect of the optogenetic activation of DRN 5-HT neurons targeted by Tph2-tTA transgenic mice in the nose-poke self-stimulation test and CPP test. (**A**) Schematic of the experiment. (**B**,**C**) Four weeks after injection, coronal sections containing the dorsal raphe nucleus (DRN) were prepared and stained by anti-green fluorescent protein (GFP) and anti-tryptophan hydroxylase (TPH) antibodies. Stained sections were imaged using confocal microscopy. Scale bars = 100 μm (low magnification), 20 μm (high magnification). Aq, Aqueduct. (**D**) The number of TPH- and GFP-immunoreactive cells in TetO::Venus injected animals were counted. (Top) Data represent mean ± SEM of the percentage of double-positive cells in GFP-positive cells. (Bottom) Data represent mean ± SEM of the percentage of double-positive cells in TPH-positive cells. *n* = 3 mice. (**E**) After injection of TetO::Venus or TetO::CheRiff in mTph2-tTA mice, the number of nose-poke responses in the operant chamber was measured. Blue light (20 Hz, 10 ms duration, 20 pulses, 5 mW) was delivered when mice performed nose-poke responses. Data represent mean ± SEM. *n* = 9 (Venus), 16 (CheRiff) mice. *** *p* < 0.001 vs. Venus (Day 1; TetO::Venus, 15.4 ± 6.5, TetO::CheRiff, 544.2 ± 83.9, Day 2; TetO::Venus, 11.8 ± 3.6, TetO::CheRiff, 660.4 ± 72.0, Day 3; TetO::Venus, 10.1 ± 2.4, TetO::CheRiff, 684.7 ± 67.5; two-way repeated-measures ANOVA; Interaction, F(2, 46) = 2.454, *p* = 0.0972, Time, F(1.972, 45.36) = 2.125, *p* = 0.1318, Opto, F(1, 23) = 44.80, *p* < 0.001, Subject, F(23, 46) = 10.34, *p* < 0.001; Bonferroni posttests; Day 1–3, *** *p* < 0.001). (**F**) In the conditioned place preference (CPP) test, spent time in the chamber associated with blue light illumination to the DRN were examined. Data represent mean + or − SEM. *n* = 6 (Venus), *n* = 7 (CheRiff) mice. ** *p* < 0.01 vs. Venus. (Venus, −16.67 ± 60.36 s, CheRiff, 230.5 ± 50.1 s; unpaired *t*-test; *p* = 0.0088). (**G**) Representative heat map of mouse movement during the pretest at Day 1 and posttest at Day 4.

To further investigate the role of DRN 5-HT neurons in reward processing, we employed a different method to target 5-HT neurons; Tph2-tTA transgenic mice which expresses tetracycline-controlled transcriptional activator (tTA) selectively in 5-HT neurons. Different from previous reports using these mice with knock-in allele bearing tTA-dependent enhancer (TetO) and transgenes (ChR2) [33,36,41], we combined these mice with AAV bearing TetO and transgenes. We immunohistochemically evaluated the specificity of transgene expression in these mice. We found that 95.5% ± 0.7% of Venus immunoreactive cells were TPH2 immunoreactive in the DRN in Tph2-tTA mice injected with AAV-TetO-Venus (TetO::Venus) (*n* = 3 mice, Figure 3A–D). Moreover, 74.5% ± 3.6% of TPH2-immunoreactive cells were also immunoreactive for Venus (*n* = 3 mice, Figure 3D). To investigate the effect of the activation of DRN 5-HT neurons on operant and Pavlovian conditioning in these mice, we performed the nose-poke self-stimulation test and CPP test (Figure 3E–G). Four weeks after injection of AAV-TetO-CheRiff-EGFP in the DRN of Tph2-tTA mice (TetO::CheRiff), they were placed in an operant chamber equipped with one active nose-poke port. We found that TetO::CheRiff mice showed significantly more nose-poke responses relative to the mice injected with TetO::Venus throughout the three days of the experiment (Figure 3E). We performed the CPP test using a different cohort of mice. After conditioning with photostimulation of DRN for two days, TetO::CheRiff mice showed significantly higher CPP scores than TetO::Venus controls (Figure 3F,G). Moreover, two-way repeated-measures ANOVA of spent time in the chamber associated with light stimulation in Days 1 and 4 revealed the significant interaction between time and AAVs (F(1, 11) = 10.10, *p* < 0.01, Supplementary Figure S1B). These results indicate that the activation of DRN 5-HT neurons targeted by Tph2-tTA mice was sufficient to elicit self-stimulation behavior and place preference.

### 2.4. Optogenetic Inhibition of DRN 5-HT Neurons and Their Terminals in the VTA Elicits Conditioned Place Aversion

**Figure 4:**
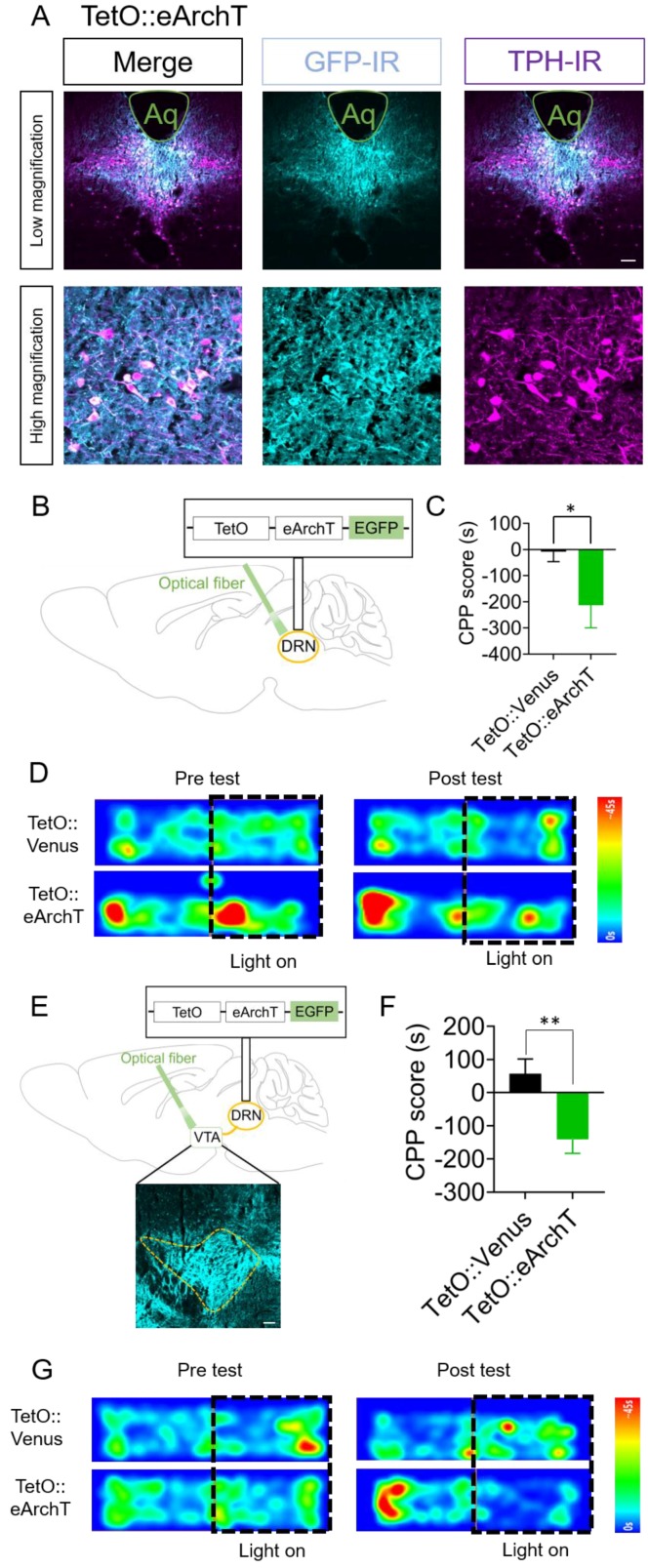
Optogenetic inhibition of DRN 5-HT neurons and their terminals in the VTA elicits conditioned place aversion. (**A**) Four weeks after injection, coronal sections containing the dorsal raphe nucleus (DRN) were prepared and stained by anti-green fluorescent protein (GFP) and anti-tryptophan hydroxylase (TPH) antibodies. Stained sections were imaged using confocal microscopy. Scale bars = 100 μm (low magnification), 20 μm (high magnification). Aq, Aqueduct. (**B**) Schematic for the injection of adeno-associated virus (AAVs) in the DRN. (**C**) After injection of TetO::Venus or TetO::eArchT in the DRN of Tph2-tTA mice, spent time in the chamber associated with green light illumination to the DRN were measured. Data represent mean ± SEM. *n* = 11 (Venus), *n* = 8 (eArchT) mice. * *p* < 0.05 vs. Venus. (Venus, −8.3 ± 38.0 s, eArchT, −213.6 ± 85.9 s; unpaired *t*-test; *p* = 0.028). (**D**) Representative heat map of mouse movement during the pretest at Day 1 and posttest at Day 4. (**E**) Schemes showing the sites of AAV injection and fiber implantation. Scale bars = 100 μm. (**F**) After injection of TetO::Venus or TetO::eArchT in the DRN, spent time in the chamber associated with green light illumination to the ventral tegmental area (VTA) were measured. Data represent mean ± SEM. *n* = 9 (Venus), *n* = 7 (eArchT) mice. ** *p* < 0.01 vs. Venus. (Venus, 57.3 ± 44.4 s, eArchT, −141.1 ± 41.7 s; unpaired *t*-test; *p* = 0.0067). (**G**) Representative heat map of mouse movement during the pretest at Day 1 and posttest at Day 4.

Finally, we investigated the effect of inhibition of DRN 5-HT neurons on aversion processing. To this end, we made AAV-TetO-eArchT-eYFP-WPRE (TetO::eArchT) [42], expressing an inhibitory optogenetic actuator activated by green light. Four weeks after injection of TetO::eArchT in Tph2-tTA mice, we tested the effect of photoinhibition of the DRN in the CPP test (Figure 4A–D). To assess aversive properties, we associated green light application on the DRN with confinement to a chamber that the mice initially preferred. After conditioning with green light application for two days, TetO::eArchT mice showed significantly lower CPP scores (i.e., spent less time in the chamber associated with light) than TetO::Venus controls at Day 4 of the CPP test (Figure 4C,D). Moreover, two-way repeated-measures ANOVA of spent time in the chamber associated with light stimulation in Days 1 and 4 revealed the significant interaction between time and AAVs (F(1, 17) = 4.476, *p* < 0.05, Supplementary Figure S1C). To determine whether this aversion was mediated by VTA-projecting 5-HT neurons in the DRN, fiber-optics were implanted into the VTA of TetO::eArchT mice. We found that TetO::eArchT mice showed significantly lower CPP scores than the control group (Figure 4E–G). Moreover, two-way repeated-measures ANOVA of spent time in the chamber associated with light stimulation in Days 1 and 4 revealed the significant interaction between time and AAVs (F(1, 14) = 10.11, *p* < 0.01, Supplementary Figure S1D). These results suggest the importance of VTA-projecting 5-HT neurons in the DRN in the processing of aversive information.

## 3. Discussion

In the present study, we investigated the effect of optogenetic manipulation of DRN 5-HT neurons on reward-related behaviors using two different strategies to target 5-HT neurons specifically; an AAV bearing the mouse TPH2 promoter [31], and Tph2-tTA transgenic mice [34]. We found that stimulation of DRN 5-HT neurons elicited reinforcement for self-stimulation behavior and induced conditioned place preference in both targeting methods. Moreover, we found that inhibition of DRN 5-HT neurons or 5-HT projection from the DRN to the VTA induced conditioned place aversion. Collectively, these results suggest that DRN 5-HT neurons projecting to the VTA are a major modulator of the balance between reward and aversion.

Lines of evidence have demonstrated that 5-HT plays a critical role in the pathophysiology and therapeutics of mental disorders including major depression and schizophrenia. In humans, drugs enhancing 5-HT neurotransmission via reuptake inhibition have been used as antidepressants [26,27,28,29]. We and others have shown that the optogenetic activation of DRN 5-HT neurons elicits antidepressant-like effects in mice and rats [31,43]. In addition to mental disorders, many reports have demonstrated that 5-HT neurons are involved in reward processing [8,9,10,11,32,33,34,35]. Some reports have shown that the activation of DRN 5-HT neurons elicits reward-related behaviors including self-stimulation and real-time place preference [9]. More recently, Wang et al. have shown that the activation of 5-HT projection from the DRN to the VTA promotes conditioned place preference [11]. In contrast, many reports have also indicated that the activation of DRN 5-HT neurons is not sufficient for producing rewarding properties [32,33,34]. Thus, the rewarding potency of DRN 5-HT neurons is currently under debate.

In this context, our data, using two different strategies targeting DRN 5-HT neurons, clearly show that the activation of DRN 5-HT neurons has rewarding properties. In contrast, it should be noted that we previously showed that the activation of DRN 5-HT neurons transduced by a lentiviral vector, not an AAV, bearing the same promoter did not induce real-time place preference [31]. Moreover, Miyazaki et al. have shown that moderate activation of DRN 5-HT neurons does not show reinforcing effects using Tph2-tTA mice also carrying a TetO-dependent ChR2 (C128S mutant)-expressing allele at their β-actin locus [33]. These discrepancies are consistent with previous reports using ePet-Cre mice; Liu et al. showed that the activation of DRN 5-HT neurons elicits strong reward-related behaviors in ePet-Cre mice, whereas McDevitt et al. showed that it does not [9,32]. Although the reason for these discrepancies is still unclear, one possible explanation is that the copy number of ChR2-expressing vectors/alleles may be a determinant of induction of reward-related behaviors. In general, the titer of lentiviral vectors is around 1 × 10^10^ /mL [31,44], which is far less than that of AAV (around 1 × 10^13^ /mL). In knock-in mice, the number of ChR2-expressing alleles is one or two, which is far less than that of AAV, although knock-in at the β-actin locus induces a high level of transgene expression [45]. Transduction of the same neurons with low copy numbers (lentiviral vectors, knock-in) failed to produce reward-related behaviors, whereas those with high copy numbers did, supporting this possibility. Taken together, these data suggest that it is necessary to examine whether the activation of DRN 5-HT neurons produces a rewarding effect when the effect of the activation of DRN 5-HT neurons on other behaviors is to be investigated. On the other hand, recent reports have demonstrated the heterogeneity in DRN 5-HT neurons from the perspective of their function as well as their anatomical connection [43]. To this end, transgene expression in this study was mainly present within −4.36 mm to −4.72 mm from the bregma, indicating that the most anterior part and most posterior part of the DRN was not efficiently infected. Therefore, it is still possible that stimulation of the most anterior or posterior part of DRN 5-HT neurons might have the opposite effect in the CPP and self-stimulation paradigm. Moreover, Liu et al. and McDevitt et al. have shown that stimulation of DRN 5-HT neurons does and does not elicit reward-related behaviors, respectively, although they used the same genetic mouse line (ePet-Cre) and AAVs. Although they reached a similar conclusion as McDevitt et al. by using SERT-Cre mice, Fonseca et al. discussed the possible involvement of AAV serotype [34]. Liu et al. used AAV2/9 whereas Fonseca et al. and McDevitt et al. used AAV2/1 [9,32,34]. The AAV-DJ we used in this study was the shuffle serotype of AAV-2, 4, 8, 9, and AAVs for other mammals [46]. Thus, it is possible that different serotypes may transduce different subpopulations of DRN 5-HT neurons.

The DRN contains several types of neurons, 5-HTergic, dopaminergic, GABAergic, and glutamatergic [47,48]. Recent studies show that some 5-HT neurons in the DRN also express vesicular glutamate transporter 3 (vGluT3) [49,50]. Histological analyses have revealed that these vGluT3-positive 5-HT neurons are mainly located within the midline area of the ventral part of the DRN, whereas vGluT3-negative 5-HT neurons are located both in the midline area and lateral wing subregions of the DRN [51,52]. Ren et al. demonstrate that 5-HT neurons in the midline area mainly project to structures including the VTA, LH and NAc [43]. Moreover, axon terminals of vGluT3-positive 5-HT neurons form excitatory synapses with dopamine neurons in the VTA [11]. Interestingly, reward-related behavior induced by the optogenetic activation of DRN 5-HT neurons is partly attenuated in Tph2 or vGluT3 deficient mice [9]. These reports indicate that vGluT3-positive 5-HT neurons in the DRN play an important role in the reward effects of the activation of DRN 5-HT neurons. Consistent with these reports, our data showed that the activation of VTA-projecting 5-HT neurons in the DRN elicited reinforcing effect and rewarding effect, although the necessity of glutamate and 5-HT has yet to be investigated. In this study, we examined the involvement of the VTA, LH, NAc, CeA, and VP. However, we cannot rule out the possibility that DRN 5-HT neurons projecting to other brain regions play a critical role in reward/aversion. For example, previous studies indicate the importance of other brain areas such as the prefrontal cortex (PFC) in drug addiction [52,53]. We have reported that the inactivation of excitatory neurons in the medial PFC inhibits the formation and retrieval of cocaine-associated memories [54]. An application of 5-HT inhibits pyramidal neurons through the activation of several types of 5-HT receptors including 5-HT_1A_ [55]. Anatomically, DRN 5-HT neurons strongly innervate the PFC [23]. Taken together, it is possible that stimulation of PFC-projecting 5-HT neurons in the DRN might inhibit the formation of CPP, although further behavioral analysis is necessary. In this study, we optogenetically activated DRN 5-HT neurons at a frequency of 20 Hz. One may think that behavioral consequences induced by this strong artificial activation are not physiological. However, previous literature have shown that natural rewards such as sucrose activates DRN 5-HT neurons [10]. Moreover, in vivo recordings in non-human primates suggest that appetitive reward increases the activity of DRN neurons from several Hz to more than 30 Hz [56]. Collectively, these data indicate that optogenetic activation at 20 Hz in this study reflects the physiological activation of DRN 5-HT neurons induced by appetitive reward.

Moreover, we found that optogenetic inhibition of VTA-projecting 5-HT neurons in the DRN was sufficient for inducing conditioned place aversion. To the best of our knowledge, this is the first report showing that basal activity of 5-HT neurons projecting from the DRN to the VTA is necessary for maintaining a balance between reward and aversion. A previous report has shown that lowering the activity of VTA dopamine neurons can induce aversive response and behavior [57]. Moreover, Solecki et al have shown that photoinhibition of VTA dopamine neurons reduced the motivation to seek cocaine [58]. Taken together with our result indicating that inhibition of 5-HT projection from the DRN to the VTA is aversive, it is possible that craving-like behavior induced by drugs of abuse may be suppressed by drugs that selectively inhibit 5-HT neurons projecting from the DRN to the VTA. In this study, molecular mechanisms underlying the reward/aversive effect induced by VTA-projecting DRN serotonin neurons is unclear. Previous literature have indicated that 5-HT2A and 5-HT2C receptors play a key role in the interaction between serotonergic neurons and mesencephalic dopaminergic neurons [21,22]. Moreover, Liu et al have electrophysiologically shown that antagonists for 5-HT2A and AMPA receptors inhibit the excitatory effect of VTA-projecting DRN serotonin neurons in VTA dopamine neurons [9]. Collectively, it is possible that the effects of serotonin and glutamate through 5-HT2A, 5-HT2C, and AMPA receptors play a key role in the reward/aversive effect. Although our data indicate the importance of VTA-projecting DRN 5-HT neurons in aversion, we cannot rule out the possibility that DRN 5-HT neurons projecting other brain regions play a critical role in aversion. To this end, further investigation using optogenetic inhibition of 5-HT axon terminals in other brain regions than the VTA is needed.

In summary, our data provide direct evidence that selective stimulation of DRN 5-HT neurons projecting to the VTA was sufficient for reinforcing effect and conditioned place preference. Furthermore, we showed that inhibition of the same neurons produced conditioned place aversion. These data indicate that the activity of DRN 5-HT neurons projecting to the VTA is a key modulator of balance between reward and aversion.

## 4. Materials and Methods 

### 4.1. Animals

All mice were handled in accordance with the ethical guidelines of the Kyoto University Animal Research Committee (Approval code: 19-41). Adult male C57BL/6J mice (8–12 weeks old, Nihon SLC, Shizuoka, Japan) and Tph2-tTA mice (8–12 weeks old, [39]) were housed in groups (no more than 6 mice in an individual cage) in a plastic cage with wooden bedding and free access to food (MF, Oriental Yeast, Tokyo, Japan) and water, and kept under constant ambient temperature (24 ± 1 °C) and humidity (55% ± 10%), with 12 h light–dark cycles. Cages were open to the ambient room. Mice were randomly assigned to each experimental group. We used male mice in this study according to a previous similar study [11].

### 4.2. Stereotaxic Surgeries

Stereotaxic surgeries were conducted using a small animal stereotaxic frame (Narishige, Tokyo, Japan) according to the Brain Atlas [59]. The animals were anesthetized with sodium pentobarbital (50 mg/kg, i.p., Kyoritsu Seiyaku, Tokyo, Japan) or 3% isoflurane (Escain, Pfizer, Tokyo, Japan). The adeno-associated virus (AAV) vectors, mTPH2::Venus, mTPH2::CheRiff, TetO::Venus, TetO::CheRiff and TetO::eArchT were microinjected at 1 µL per mouse into the DRN (AP –4.3 mm, ML +1.2 mm, depth +3.6 mm at 20° from the bregma,). For behavioral experiments, 4–5 weeks after viral injection the animals were implanted with a fiber-optic cannula, where the tip of the cannula was placed just above the dorsal border of the target regions. The following coordinates were used for fiber implantation, DRN (AP –4.3 mm, ML +1.2 mm, DV +3.3 mm at 20° from the bregma), VTA (AP –3.1 mm, ML ±0.7 mm, DV +4.3 mm from the bregma), LH (AP –2.5 mm, ML ±0.9 mm, DV +4.8 mm from the bregma), CeA (–1.1 mm, ML ± 2.5 mm, DV +4.3 mm from the bregma) NAc (AP +1.5 mm, ML ±0.75 mm, DV +3.9 mm from the bregma), and VP (AP +0.3 mm, ML ±1.3 mm, DV +4.6 mm from the bregma). A fiber-optic cannula was unilaterally implanted for optogenetic manipulation of axon terminals. The experimenters who carried out behavioral analyses were blinded to the group allocation. After behavioral analyses, all mice were sacrificed, and the AAV infection was verified immunohistochemically. Data points obtained from mice with failed AAV vector infection were excluded (5 mice were excluded by this criterion throughout the study).

### 4.3. Vector Construction

For construction of AAV-mTPH2-Venus-WPRE (mTPH2::Venus), the mTPH2-Venus fragment was amplified by PCR from pTYF-mTPH2-Venus-WPRE [31], and ligated to AAV backbone obtained from pAAV-hSyn-DIO-hM3Dq-mCherry (Addgene, 44361). For construction of AAV-mTPH2-CheRiff-EGFP-WPRE (mTPH2::Cheriff), the Cheriff-EGFP fragment was PCR amplified from DRH296: FCK-Optopatch2 (Addgene, 51694), and ligated to mTPH2::Venus. For construction of AAV-TetO-Venus (TetO::Venus), the TetO fragment amplified by PCR from pFUW-TetO-OSKM (Addgene, 20321) and the Venus fragment amplified by PCR from pTYF-mTPH2-Venus-WPRE [31,60] were ligated to the AAV backbone obtained from pAAV-hSyn-DIO-hM3Dq-mCherry (Addgene, 44361). For construction of AAV-TetO-CheRiff-EGFP (TetO::CheRiff), the CheRiff-EGFP fragment was PCR amplified from DRH296: FCK-Optopatch2 (Addgene, 51694), and ligated to TetO::Venus. For construction of AAV-TetO-eArchT-EYFP (TetO::eArchT), the eArchT-EYFP fragment was PCR amplified from mTPH2-eArchT3.0-eYFP-WPRE [34], and ligated to TetO::Venus.

### 4.4. Production and Purification of Adeno-Associated Virus (AAV) Vector

Lenti-X 293T cells (Clontech, Mountain View, CA, USA) were grown to 60%–70% confluency, and 8 µg of pHelper, 5 µg of pAAV-DJ, and 5 µg of transfer plasmid were transfected with polyethylenimine (Polysciences, Warrington, PA, USA). After 60–72 h of incubation, the supernatant was aspirated and 400 µL of 1× Gradient Buffer was added to the cells on each plate, and then collected. The cell suspension was frozen in liquid nitrogen for 10 min, and placed in a 55 °C water bath until the cells were completely thawed. After lysis, they were triturated by using a 20 mL syringe and a 23-gauge needle (Terumo, Tokyo, Japan). This freeze-thaw cycle was repeated 3 times. After the addition of 1 µL of benzonase (Sigma-Aldrich, St. Louis, MO, USA), the lysate was incubated at 37 °C for 45 min, centrifuged for 15 min at 3000 × *g* in R20A2 rotor (Koki Holdings, Tokyo, Japan), and then the supernatant was collected. A discontinuous density gradient of 15%, 25%, 40%, and 58% iodixanol was prepared in an ultracentrifuge tube, and the supernatants were dripped onto the top layer of the density gradient. The tube was ultracentrifuged at 48,000 rpm, 18 °C for 1 h 45 min in a 50.2Ti rotor (Beckman-Coulter, Brea, CA, USA). After ultracentrifugation, a 5 mL syringe with an 18-gauge needle was inserted approximately 1–2 mm below the boundary surface between 40% and 58% gradient buffer layers, and 3 mL of solution was slowly extracted. This was aliquoted and stored at −80 °C until use. The titer of AAV was measured by qPCR and estimated to be about 1.0 × 10^13^ vg/mL.

### 4.5. In Vivo Optogenetic Manipulation

Fiber-optic cannulae were made of multimode LC/PC ceramic ferrules (1.25 mm outer diameter, 270 µm hole size, Thorlabs, Newton, NJ, USA) and plastic optic fiber (CK10, 250 µm diameter, NA 0.5, Mitsubishi Rayon, Tokyo, Japan). The fiber-optic cannula implanted to mice were connected to the fiber-optic patch cord (M83L01, Thorlabs) or bifurcated fiber bundle (BFYL2LF01, Thorlabs) coupled with the rotary joint (Doric Lenses, Québec, QC, Canada). Light emitted from the diode-pumped solid-state (DPSS) laser (Beijing Viasho Technology, Beijing, China) was converged to the fiber-optic which was connected to the rotary joint. The DPSS laser was driven by the electric stimulator (Nihon Kohden, Tokyo, Japan). For mTPH2::CheRiff, mTPH2::Venus, TetO::CheRiff, and TetO::Venus mice, blue light illumination was delivered when animals nose-poke in self-stimulation test (soma stimulation 473 nm, 5 mW at the tip of the fiber, 20 Hz frequency, 10 ms duration, 20 pulses; unilateral axon terminal stimulation 473 nm, 1–5 mW at the tip of the fiber, 20 Hz frequency, 10 ms duration, 20 pulses) or throughout the conditioning session of conditioned place preference test (soma stimulation 473 nm, 5 mW at the tip of the fiber, 10 ms duration, 20 Hz frequency, 20 s on/10 s off; unilateral axon terminal stimulation 473 nm, 1–5 mW at the tip of the fiber, 20 Hz frequency, 10 ms duration, 20 pulses). For TetO::eArchT and TetO::Venus mice, green light illumination (soma stimulation 532 nm, 2–2.5 mW at the tip of the fiber, continuous; unilateral axon terminal stimulation 532 nm, 2–2.5 mW at the tip of the fiber, continuous) was delivered throughout the conditioning session of the conditioned place aversion tests.

### 4.6. Behavioral Tests

All behavioral tests were performed and analyzed by experimenters who were blind to the injected AAV. Animals with misplaced fiber-optic cannula were excluded from analyses (4 mice were excluded by this criterion throughout the study). Inclusion criteria for the place of fiber-optic tips is following; the tip of fiber-optic was located in or on the border of each brain area (DRN, Bregma −4.36 to −4.72; VTA, Bregma −3.16 to −3.08; LH, Bregma −2.54 to −2.18; CeA, Bregma −1.94 to −0.58; NAc, Bregma +0.62 to +1.94; VP, Bregma −0.34 to +0.86 in mm). All behavioral tests were performed during the light phase of the day cycle.

### 4.7. Self-Stimulation in an Operant Chamber

The operant conditioning chamber (dimensions: 15.24 × 13.34 × 12.7 cm, L×W×H; Med Associates, Fairfax, VT, USA) was equipped with a nose-poke port (ENV-303M; Med Associates) and encased in a sound-attenuating box. Nose-poking through the hole resulted in the delivery of blue light pulses into the target region through the optical fiber. Twenty pulses were delivered for each nose-poking response. Animals were plugged into the fiber-optic patch cord and placed into the chamber and subsequently allowed to self-stimulate the target nuclei for 30 min. The mice performed the task for 3 consecutive days.

### 4.8. Conditioned Place Preference (CPP) Test

The CPP test was performed according to previous reports [54,61]. The CPP apparatus consisted of two equal-sized compartments (dimensions: 15 × 24 × 30 cm) with distinct tactile and visual cues. One compartment was white with a textured floor and the other one was black with a smooth floor. In pretest sessions on Day 1, mice without the fiber patch cord were allowed to explore two compartments freely for 900 s, and the time spent in each compartment during the exploration period was measured using ANY-MAZE software (ANY-maze version 6.0, Stoelting, Wood Dale, IL, USA). Mice that spent more than 80% (>720 s) of the total time in one compartment in the pretest were excluded from the following procedures (9 mice were excluded by this criterion throughout the study). We used a bias-like protocol [62] and designated the compartment in which each mouse spent less time (<450 s) in the pretest as the stimulation-paired compartment for that animal. In total, 20 mice were associated with light and white chamber, while 11 mice were associated with light and black chamber. On Days 2 and 3 (conditioning), mice were connected to the fiber-optic patch cord and confined to a non-stimulation-paired compartment for 20 min without light delivery. After at least 4 h, each mouse was connected to the fiber-optic patch cord and confined to the stimulation-paired compartment with light delivery for 20 min. On Day 4 (posttest), mice without the fiber patch cord were allowed to freely explore the two compartments for 900 s, and the time spent in each compartment during the exploration period was measured. The CPP scores were calculated by subtracting the time spent in the light-paired compartment during the pretest from that during the posttest.

### 4.9. Conditioned Place Aversion (CPA) Test

The same apparatus was used as the CPP test. After the same pretest as the CPP test, we designated the compartment in which each mouse spent more time (>450 s) in the pretest as the inhibition-paired compartment for that animal. In total, 12 mice were associated with light and white chamber, while 23 mice were associated with light and black chamber. On Days 2 and 3 (conditioning), each mouse was confined to the non-inhibition-paired compartment without light delivery for 20 min. After at least 4 h, each mouse was confined to the inhibition-paired compartment with light delivery for 20 min. The CPA scores were calculated by subtracting the time spent in the light-paired compartment during the pretest session from that spent in the same compartment during the posttest.

### 4.10. Histology

The animals were deeply anesthetized with pentobarbital and transcardially perfused with PBS followed by 4% paraformaldehyde (Nacalai Tesque, Kyoto, Japan) in PBS. After perfusion fixation, the brains were harvested, equilibrated in 15% sucrose in PBS overnight and frozen. The brains were cryosectioned into 30 µm-thick coronal sections with the cryostat (Leica CM3050S; Leica Biosystems, Nussloch, Germany) and stored at −80 °C until immunohistochemical processing. For immunohistochemistry, the sections including DRN were immersed in 0.25% Triton-X 100 (Nacalai Tesque) for permeabilization and then incubated overnight at 4 °C with rabbit polyclonal anti-green fluorescent protein (GFP) antibody (1:2000; A-11122, Life Technologies, Carlsbad, CA, USA) and sheep polyclonal anti-tryptophan hydroxylase (TPH) antibody (1:200; AB1541, Merck Millipore, Burlington, MA, USA), followed by incubation with Alexa Fluor 488-labeled donkey anti-rabbit IgG (1:200; Life Technologies) and Alexa Fluor 594-labeled donkey anti-sheep IgG (1:200; Life Technologies) for 2 h at room temperature. The antibody used for immunostaining sections including the other regions was anti-GFP antibody only. The sections were then washed in PBS and mounted on glass with Fluoromount/Plus (Diagnostic Biosystems, Pleasanton, CA, USA). Immunofluorescence was visualized using a laser scanning confocal microscopy (Fluoview FV10i, Olympus, Tokyo, Japan) with software (FV10i-SW, Olympus, Tokyo, Japan; Image J, NIH, Bethesda, MD, USA). Quantification of GFP-positive neurons, TPH-positive neurons, and double-positive neurons was performed in one section per animal with most Venus fluorescence.

### 4.11. Statistical Analysis

Statistical analysis was performed using GraphPad Prism 8 (GraphPad Software Inc., La Jolla, CA, USA). Two-sided unpaired Student’s *t*-test was used for comparisons of two individual groups unless otherwise stated. Two-way repeated-measures ANOVA with Geisser–Greenhouse correction followed by Bonferroni post hoc test was used for group comparisons unless otherwise stated. The difference was considered significant at *p* < 0.05. 

## Supplementary Materials

Supplementary materials can be found at https://www.mdpi.com/1422-0067/21/6/2160/s1.
