# Supplementary material for: The Role of Dorsal Raphe Serotonin Neurons in the Balance between Reward and Aversion
Source: Int J Mol Sci. 2020 Mar 21;21(6):2160. doi: 10.3390/ijms21062160 (PMC7139834; doi:10.3390/ijms21062160)
Supplement: Supplementary file 1 [file ijms-21-02160-s001.zip › Supplementary Figures.pdf]

Supplementary Figures

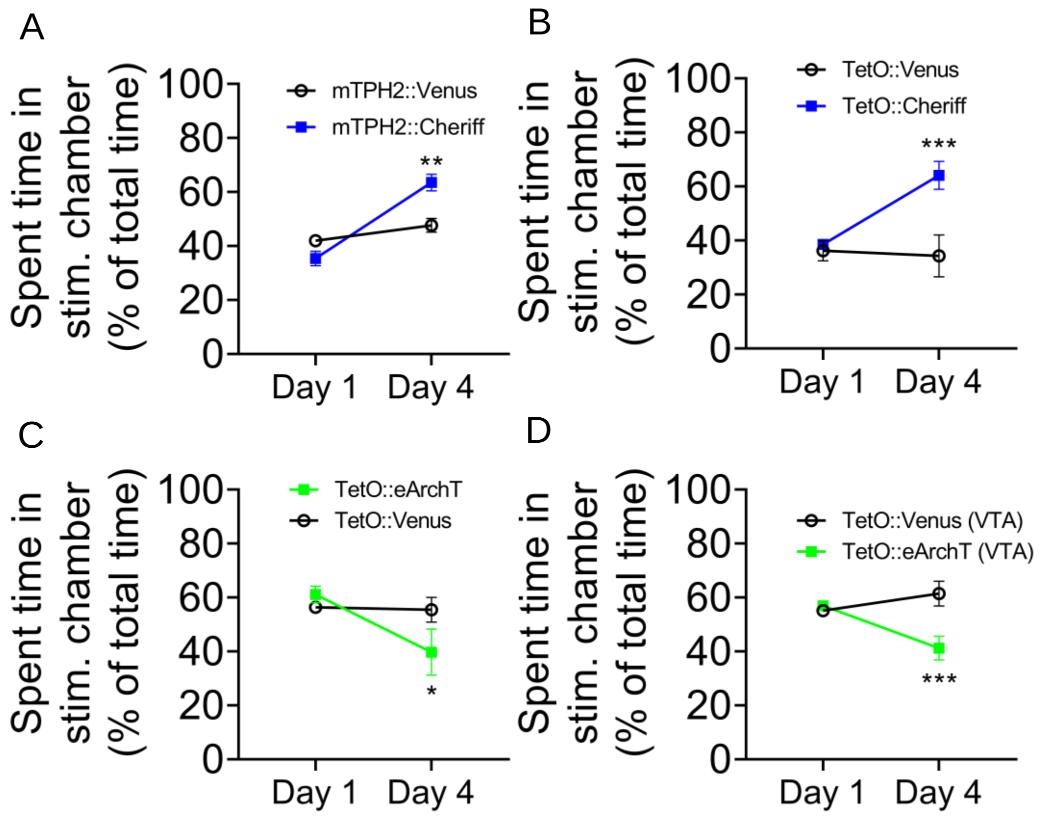

Supplementary Figure S1. Normalized spent time in the chamber associated with light stimulation in CPP and CPA tests. Spent time in the chamber associated with light stimulation normalized to total time (900 s) in pretest (day 1) and posttest (day 4) was calculated. (A) the same mice as shown in Fig. 1F, (B) the same mice as shown in Fig. 3F, (C) the same mice as shown in Fig. 4C, (D) the same mice as shown in Fig. 4F. Data represents mean ± SEM. $^{*}P < 0.05$, $^{**}P < 0.01$, $^{***}P < 0.001$ vs. Venus.

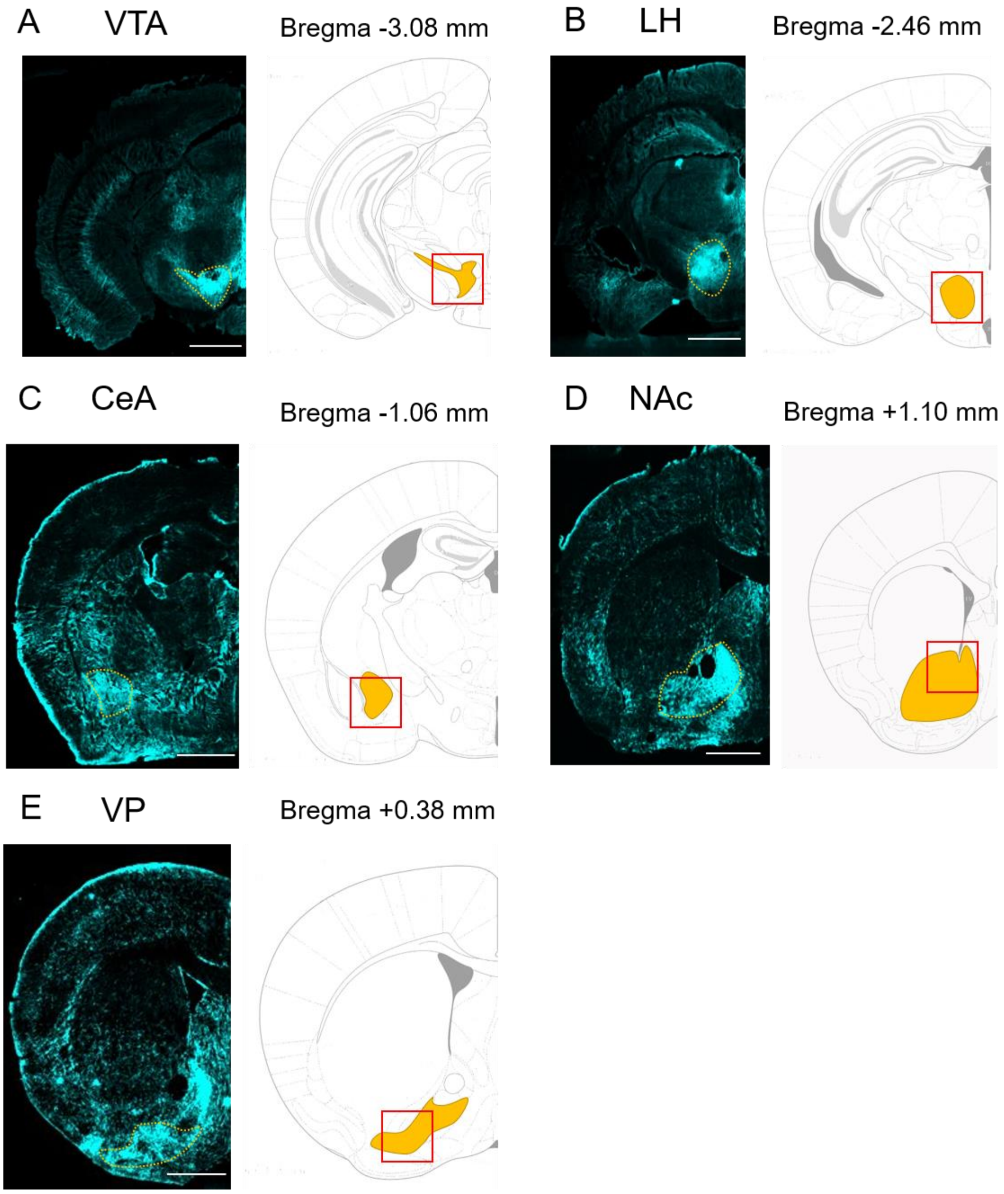

Supplementary Figure S2. Wide-field view of DRN 5-HT projection in target areas. Four weeks after mTPH2::CheRiff injection in the DRN, coronal sections containing the VTA (A), LH (B), CeA (C), NAc (D), and VP (E) were prepared and stained by anti-GFP antibodies. Stained sections were imaged using confocal microscopy. Scale bars = 1 mm (Left). Drawings of coronal sections including projection area from the Atlas. Each projection area is colored in orange. Red frames indicate the position of high magnification image shown in Fig. 2 (Right).

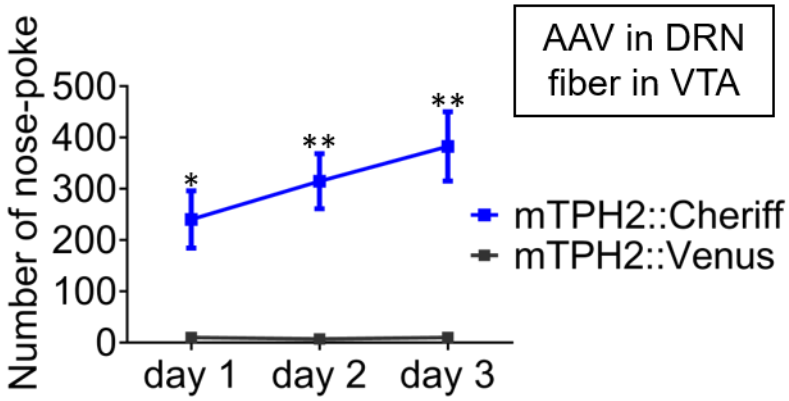

Supplementary Figure S3. Blue light delivery into the VTA of mTPH2::Venus injected mice failed to induce self-stimulation behaviors. After injection of mTPH2::CheRiff or mTPH2::Venus, the number of nose-poke responses in the operant chamber was measured. Blue light (20 Hz, 10 ms duration, 20 pulses, 5 mW) was delivered when mice performed nose-poke responses. Data represents mean ± SEM of the number of nose-poke during 30 min session. CheRiff; n = 8 mice, Venus; n = 7 mice. $^{*}P < 0.05$, $^{**}P < 0.01$ vs. Venus.
